# Binary phase masks for easy system alignment and basic aberration sensing with spatial light modulators in STED microscopy

**DOI:** 10.1038/s41598-017-15967-5

**Published:** 2017-11-16

**Authors:** André Klauss, Florian Conrad, Carsten Hille

**Affiliations:** University of Potsdam, Institute of Chemistry, Potsdam, D-14476 Germany

## Abstract

The use of binary phase patterns to improve the integration and optimization of spatial light modulators (SLM) in an imaging system, especially a confocal microscope, is proposed and demonstrated. The phase masks were designed to create point spread functions (PSF), which exhibit specific sensitivity to major disturbances in the optical system. This allows direct evaluation of misalignment and fundamental aberration modes by simple visual inspection of the focal intensity distribution or by monitoring the central intensity of the PSF. The use of proposed phase masks is investigated in mathematical modelling and experiment for the use in a stimulated emission depletion (STED) microscope applying wavefront shaping by a SLM. We demonstrate the applicability of these phase masks for modal wavefront sensing of low order aberration modes up to the third order of Zernike polynomials, utilizing the point detector of a confocal microscope in a ‘guide star’ approach. A lateral resolution of ~25 nm is shown in STED imaging of the confocal microscope retrofitted with a SLM and a STED laser and binary phase mask based system optimization.

## Introduction

STED microscopy relies on beam shaping of a high intensity laser to create a focus featuring a central intensity zero surrounded by high intensity lobes^1–3^. This STED point spread function (PSF) is brought to an exact overlap with the diffraction-limited spot exciting fluorescence in a confocal microscope. At sufficient high STED intensity at a wavelength red-shifted to the excitation laser, excited molecules in the periphery of the excitation spot are forced to the ground state by stimulated emission. Hence, the effective PSF, from where fluorescence emission can be detected, is diminished to diffraction-unlimited size defined by the STED PSF zero, where stimulated emission is not saturated.

Liquid crystal phase-only spatial light modulators (SLM), realizing freely programmable diffractive optical elements, have been used for STED laser beam shaping commonly^4,5^. Compared to a static vortex phase plate the programmable device increases cost and complexity of the STED setup. On the other hand, the flexibility is won to apply either the vortex phase mask (PM) for ‘doughnut’ PSF creation and lateral confinement of fluorescence or the circular PM for creation of the ‘optical bottle’ PSF and axial fluorescence confinement, or both in parallel^6,7^. Another crucial advantage of the adaptive optical element is that system aberration can be corrected for by the same SLM used for beam shaping. This is achieved by application of distortion-compensating PMs on the SLM^6^ to flatten the resulting wavefront and counter the critical sensitivity of ‘doughnut’ and ‘optical bottle’ PSFs regarding aberration and misalignment^8–10^. Lateral PM alignment by phase pattern shifting on the SLM display and the application of tilt phases to achieve holographic and thus contact-free fine tuning of the STED beam may be seen as an additional benefit of the SLM in the setup^11^.

Precise alignment of a microscope’s optical path is critical to realize diffraction-unlimited imaging. Direct observation of the focus is common means for this task. In STED setups, especially the ‘doughnut’ or the ‘optical bottle’ are observed and their symmetry is optimized by fine alignment of the STED laser beam path^5,8^. Problems arise here, if system aberration due to imperfect surfaces of optical components are present. It has been noted e.g. that PM misalignment and low-order aberrations have similar effects on the STED focus shape^8^, making this correction modality based on PSF deformation error-prone.

To benefit in the alignment process just by visual PSF inspection from the SLM’s beam shaping capacities, we have designed and investigated binary phase patterns that create focal light distributions with distinct response features to misalignment or aberrations.

In a STED microscope, typical system aberrations such as astigmatism or coma degrade the imaging performance of the systems mostly by disturbing the intensity distribution of the ‘doughnut’ PSF crest^10^. Thus, important part of the optimization of the SLM-STED setup is the determination of the system aberration remaining after optimal alignment, in order to apply the corresponding aberration compensating PM on the SLM. Low-order aberrations that are often dominating in microscopic systems^12^ have characteristic effects on the ‘doughnut’ PSF, which are listed in literature^13,14^. If such characteristic deviations are recognized in the ‘doughnut’ PSF they can be used to identify specific aberrations by visual inspection of the PSF shape. Subsequently, these aberrations can be corrected by applying corresponding biased Zernike polynomials.

However, with an increasing amount of Zernike polynomials necessary to describe the aberrated wavefront, visual inspection of the ‘doughnut’ PSF becomes increasingly non-trivial. The combined effect of only two or three Zernike aberrations on the doughnut shape makes identification of individual contributions difficult, even for the trained eye (Supplementary Fig. S1). Here, we present alternative focal light distributions created by application of binary PMs on the SLM. These PSFs allow to decide from a single look at a 2D-PSF image if one of the low-order aberrations astigmatism, coma or trefoil is present in the wavefront, making sensorless SLM correction of basal system aberration by visual PSF inspection straight forward.

More advanced setups for adaptive optics microscopy have been realized with direct wavefront sensing applying an external Shack-Hartmann (SH) wavefront sensor^15,16^. For indirect (modal) wavefront sensing without external sensor, sets of test aberration modes are generated by an adaptive optical element and a quality measure in resulting image series is monitored^6,12,17^. Another powerful method for sensorless *in situ* wavefront correction is holographic pupil segmentation^18,19^ where single beamlets originating from different pupil segments are created one at a time by a SLM. The wavefront is reconstructed from consecutively measuring the displacement of each beamlet^18^ or by measuring the phase offset in each segment by interference with the beamlet from the centre of the pupil^19^.

Alternatively, phase retrieval methods based on the iterative Gerchberg-Saxton algorithm may be applied to estimate aberration in the pupil function from a reduced number of 2D-PSF images^20,21^ expanded by e.g. positive and negative defocus and astigmatism^16^. The high susceptibility of the Laguerre Gaussian ‘doughnut’ mode to azimuthal aberrations has been taken advantage of to realize iterative phase retrieval from even a single 2D-PSF image^22^.

Obviously not being competitive to external wavefront sensors in measuring complex aberrations, we propose the use of our binary PMs as a tool for simple and fast every day test for system aberration (e.g. after realignment) by focus inspection, avoiding the complexity of implementing an external wavefront sensor or running phase retrieval algorithms. Compared to the proposed method, the application of the pupil segmentation method may for the use of only a few segments yield better reproduction of a complex wavefront at a similar amount of necessary measurements. For systems that benefit from correction of low order aberrations, however, the binary PMs deliver a more direct feedback without having to scan the whole pupil. This may be desirable when interactively optimizing a STED beam or when problems occur with low signals due to the reduced numerical aperture of each beamlet in the pupil segmentation method.

Based on monitoring the central intensity of yielding PSFs in a ‘guide star’ approach^12^ by the point detector of a confocal system, we demonstrate the use of binary PMs in modal sensing of low-order aberrations and show that the method is well suited (but not limited to) the optimization of an SLM-STED microscope. Sharing the common feature of central zero intensity in the non-aberrated system, the ‘sensor’ PSFs may reduce light stress to the ‘guide star’ during alignment and aberration testing. Major drawback is the limitation to the low-order aberrations astigmatism, coma, and trefoil.

## Results and Discussion

Exact alignment of an optical system with unknown aberration is not straight forward. In the case of STED microscopy, this concerns the distinction between distortions of the observed intensity PSF caused by imperfect lateral positioning of the STED PM and by aberrations due to imperfect surfaces of mirrors, lenses and the SLM itself.

### Binary phase masks for beam alignment

**Figure 1:**
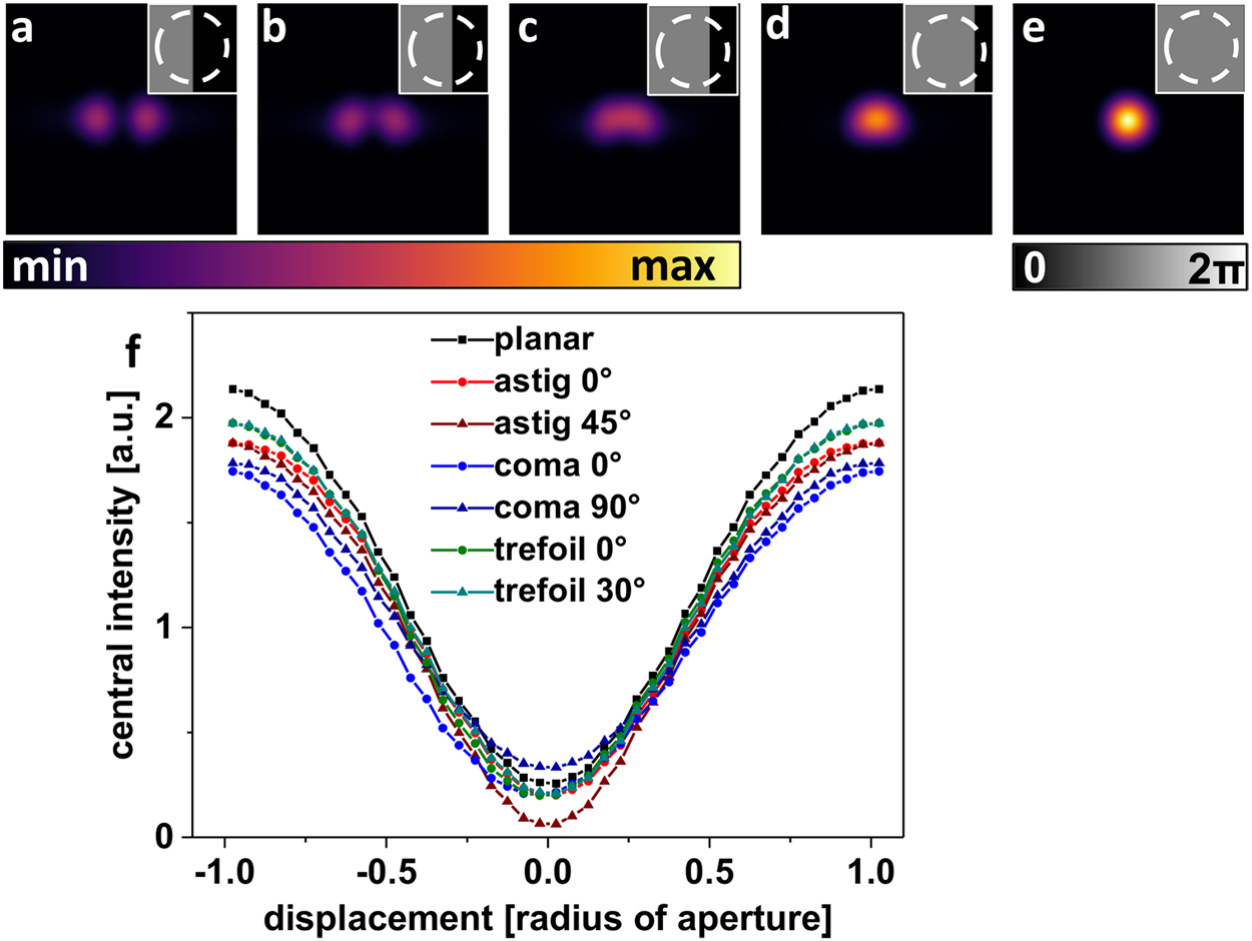
Lateral phase mask alignment: Simulations of focal light intensities resulting from phase modulation with a PM featuring a ‘half space’ π-phase step. (**a**–**e**) Focal intensity resulting from PMs shown as insets in grey scale. Dashed white circles indicate the fix position of the laser beam. (**f**) Values of the central pixel in intensity distributions like those shown in (**a**–**e**) as a function of the PM beam displacement. The central intensity of a PSF resulting from a non-aberrated wavefront is compared to the corresponding intensities resulting from wavefronts distorted by 0.4π rad astigmatism, 0.4π rad coma, or 0.4π rad trefoil aberration.

A plane wavefront with Gaussian intensity profile will focus to a diffraction-limited spot in an idealized optical system. Simulated intensity PSFs in Fig. 1 show, how imprinting a 1π rad phase step in form of a ‘half space’ phase pattern into the wavefront results in destructive interference and thus, a split PSF with a central intensity valley (Fig. 1a). Increasing lateral displacement of the phase border relative to the centre of the pupil intensity profile (indicated by white circle in Fig. 1) leads to an increasing filling of the central PSF zero (Fig. 1a–c) and finally its complete disappearance (Fig. 1e). This is easily understood as complete destructive interference can only occur if the widened intensity profile of the laser beam is divided by the phase mask into halves of equal intensity.

The lateral position of the PSF in the image plane is not influenced, however, by the shifting of the PM border through the laser intensity profile. This is of great advantage when monitoring the changes in the PSF due to PM shifting with a point detector, e.g. in a confocal detection scheme.

The characteristics of the split PSF allow determining the exact central position of the widened laser intensity profile on the SLM display. Either one acquires online the resulting PSF while shifting the phase border on the SLM display in perpendicular directions. Or one monitors only the central PSF intensity such as in systems that are not equipped with sensitive cameras or fast galvanometer beam scanners. The minimal step size for lateral shifting of the phase border position on the SLM display is the physical pixel width, 8 µm for our SLM, which allows to determine the pixel coordinates, where STED PMs should be displayed on the SLM very precisely for the pre-aligned laser beam.

Important is the finding that additional aberrations do not have a perceptible influence on the occurrence of the deepest PSF valley for central division of the beam profile by the π phase step. Related values of light intensity in the central valley of the split PSF as function of the PM displacement are given in Fig. 1f for chosen low-order aberrations. All curves show an intensity minimum for zero-displacement between pupil and phase border, assuring that position sensing by shifting a ‘half space’ phase pattern does not critically depend on low-order aberrations and thus, is suited for holographic PM alignment in systems aberrated to a certain level.

The residual central PSF intensity at zero displacement, present in all curves in Fig. 1f, is explained by the circular polarization. As a consequence, there is a polarization component perpendicular to the phase border, and focusing with an objective lens of high numerical aperture (NA) will create an axial light component^23^ that is not deleted by destructive interference.

### Binary phase masks for aberration sensing and correction

**Figure 2:**
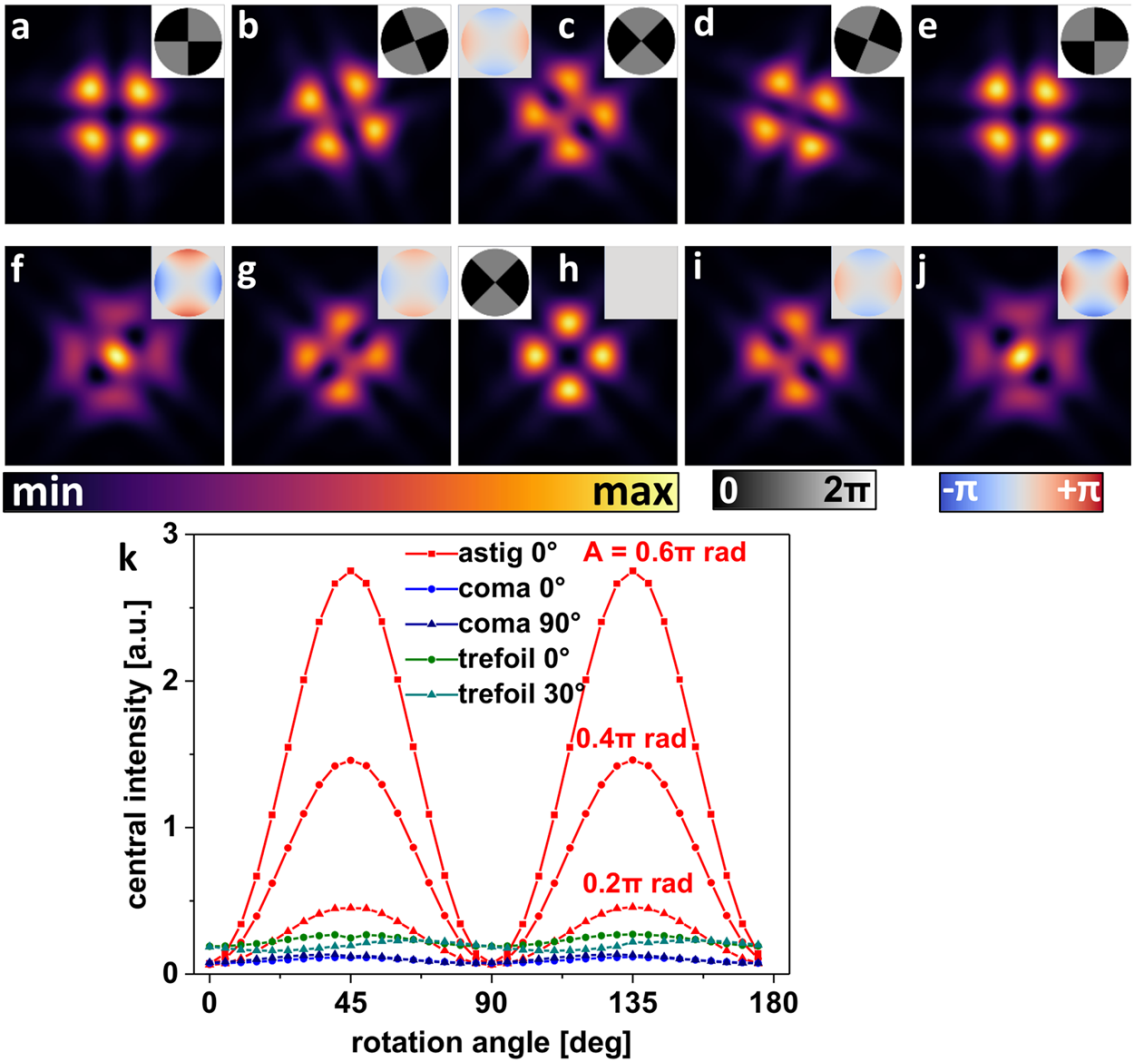
Simulations of focal light intensities of a wavefront phase modulated by a ‘four segments’ pattern. (**a**–**e**) PSF of a wavefront aberrated with 0.4π rad astigmatism (left inset in c) and modulated with the ‘four segments’ PM at different rotation angles 0°, −22.5°, −45°, −67.5°, and −90° (see insets). (**f**–**j**) Simulated PSFs of a wavefront aberrated with astigmatism of varying strength (right insets) and modulated by the ‘four segments’ PM at an angle of 45° (left inset in **h**). (**k**) Central intensities of the PSF shown above, as a function of the rotational angle of the PM and with different low-order aberration modes present in the wavefront (bias amplitude of 0.4π rad unless stated otherwise).

Following the concept of working during STED microscope optimization with a PSF that shows specific, easy to recognize changes upon misalignment, we looked for a PSF with similar properties concerning astigmatism aberration. A PM suited for astigmatism sensing by simple observation of the PSF is the ‘four segments’ PM with alternating phase values zero and π rad in the segments (Fig. 2a inset). Due to destructive interference along the segment borders, focusing of an accordingly phase-modulated laser beam results in a focal intensity distribution featuring a central minimum (Fig. 2a, h). Additional astigmatism in the wavefront, however, causes a residual central PSF intensity, depending on the rotational orientation of PM segments relative to the astigmatic wavefront deviation (Fig. 2c left inset). This residual intensity becomes maximal for a maximal overlap of astigmatic wavefront deviations and sensing segments (Fig. 2c) and minimal at a ± 45° rotation of the ‘four segments’ PM (Fig. 2a, e). Figure 2k (red) illustrates the dependence of the central PSF intensity on the rotational angle of the introduced sensing PM for three different amounts of astigmatism in the wavefront.

The rotation angle dependence of central PSF intensity due to coma (blue) or trefoil (green), shown in Fig. 2k, indicate that cross-sensitivities to other low-order aberrations are small. Corresponding signal modulations are almost two orders of magnitude smaller than the signal caused by astigmatism. Simulations show that the cross-sensitivity to trefoil aberration is a pure polarization effect leading to modulation of the axially polarized intensity component if focused by a high NA objective. Cross-sensitivity will thus be even less of concern for low NA systems.

When optimizing a STED depletion focus with an adaptive optical element, the most basic approach is to sequentially add different test aberrations of varying strength, searching to improve the rotational symmetry of the resulting ‘doughnut’ PSF. Even though the ‘doughnut’ PSF shows characteristic deformations as response to low-order aberrations, already for a limited number of Zernike polynomials the identification of individual contributions becomes a demanding task, as the combination of only two or three aberration modes results in complex shape variations of the doughnut (see Supplementary Fig. S1). The use of the proposed ‘four segments’ PSF may help to reduce the complexity. It provides the user a simple and direct feedback in form of central PSF intensity for the presence of astigmatic aberration. As the feedback signal appears on top of an almost zero background in the central PSF valley, it is well detectable.

The lateral orientation of astigmatism in the system is simply determined by observing the central intensity of the ‘four segments’ PSF while rotating the corresponding pattern on the SLM. For the orientation angle of maximal signal, compensating bias astigmatism may now be added to the PM (oriented accordingly), in order to minimize the central PSF intensity.

**Figure 3:**
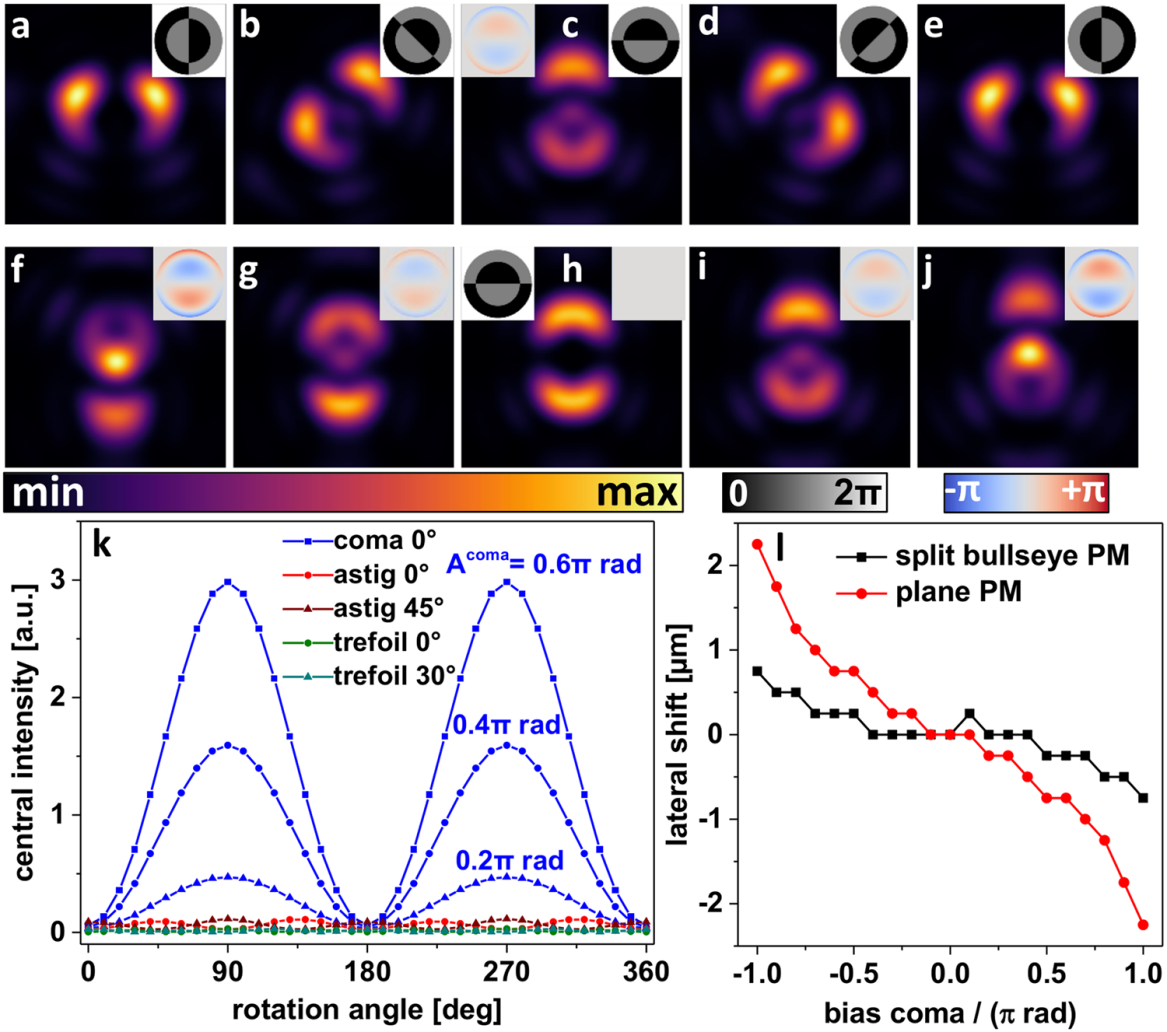
Simulations of focal light intensities of coma-aberrated wavefronts with imprinted ‘split bullseye’ PM. (**a**–**e**) calculated PSFs for coma aberration at an orientation as indicated in the left inset in (**c**) and ‘split bullseye’ PM at orientations of 0°, −45°, −90°, −135°, and −180° (right-hand side insets). (**f**–**j**) Simulated PSFs created by the ‘split bullseye’ PM with different amounts of additional coma aberration (−0.8π rad to + 0.8π rad, see right-hand side insets). (**k**) Central PSF intensity as a function of the orientation angle of the PM and in presence of additional aberration modes (bias amplitudes of 0.4π rad unless stated otherwise). (**l**) Lateral shift of the PSF generated by a plane and a ‘split bullseye’ PM as function of the bias amplitude of coma aberration.

The described method of PM-based astigmatism sensing was transferred to other low-order aberration modes as coma and trefoil. In Fig. 3, the principle idea for coma sensing is outlined. A ‘split bullseye’ PM with zero and π rad phase values (Fig. 3a inset) was designed to create a central PSF intensity zero when imprinted into a plane wavefront. Additional coma aberration will create residual central PSF intensity, depending on the orientation of the PM (Fig. 3a–e). The simulated rotational angle dependencies are given in Fig. 3k. Cross-sensitivities with the other Zernike modes under investigation are negligible for this coma-sensing PSF. Supplementary Fig. S2 demonstrates the clear perceptibility of coma (horizontally oriented) by central intensity in the engineered PSF. Interestingly, the ‘split bullseye’ PM decreases lateral PSF shift due to wavefront expansion by bias coma aberration as it is applied in modal wavefront sensing. In a certain amplitude range (−0.5π to 0.5π rad), compensation of an coma-induced lateral PSF shift by additional tilt phases^6^ becomes obsolete (Fig. 3l), reducing the complexity of modal coma sensing.

**Figure 4:**
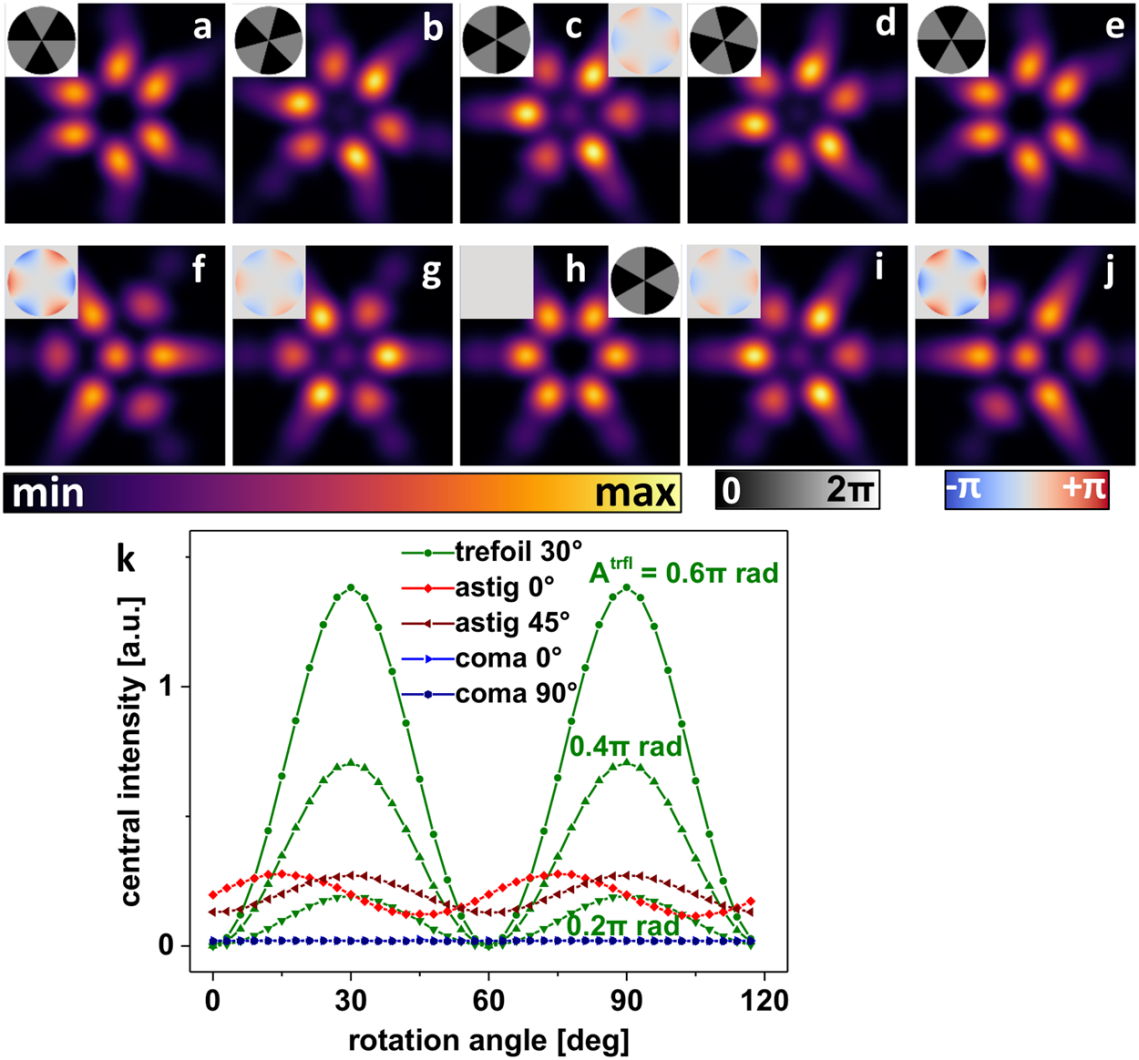
PSF simulations of trefoil-aberrated wavefronts when modulated by a ‘six segments’ PM. (**a**–**e**) orientation angle dependence of the PSF resulting from the ‘six segments’ PM. (**f**–**j**) Dependence of the ‘six segments’ PSF on trefoil aberration of different strength. (**k**) Central intensities of the ‘six segments’ PSF as function of the rotation angle of the PM and for different low-order Zernike aberration modes (bias amplitude of 0.4π rad unless stated otherwise).

The straight forwardly deduced PM for trefoil sensing is a ‘six segments’ pattern of alternating zero and π rad phase values (Fig. 4a inset). The central PSF intensity appearing in a trefoil-aberrated system as a function of PM rotation angle is depicted in Fig. 4a–e and k. A certain cross-sensitivity to astigmatism is found to also cause signal modulation when rotating the ‘six segments’ PM (reddish data points in Fig. 4k). It can be seen that this cross-sensitivity again is an effect of polarization creating axially polarized focal intensity due to high NA focusing. The cross-sensitivity will therefore disappear in low NA systems. To avoid complications with high NA objectives, we propose astigmatism sensing and correction before applying the ‘six segments’ PM trefoil sensor.

Application of the three presented PMs allows to determine orientation and strength of astigmatism, coma and trefoil by simple PSF observation and gives a straight forward guidance for STED PSF optimization as long as correction for those low-order aberrations is sufficient.

### Modal wavefront sensing with binary phase masks

Modal wavefront sensing in adaptive optics comprises expanding the aberrated wavefront successively with a set of test aberration modes and optimize a feedback signal, e.g. the maximum intensity of a probe in confocal detection^12,24^. The residual central intensity of PSF valleys formed by the binary PMs can also be used as a feedback signal for modal sensing of low-order aberrations. We see a potential advantage in the application of our binary PMs derived from the rotational sensitivity of the central PSF intensities to aberration: this allows to directly estimate the polar orientation of astigmatism, coma or trefoil in the wavefront. All three aberrations are normally described by a linear combination of two linearly independent Zernike modes (e.g. Z_3_: primary astigmatism at 45°, Z_5_: primary astigmatism at 0°). Thus, modal aberration testing in the conventional way requires the variation of the amplitude of both polynomials of each classical aberration. In the here proposed scheme applying the ‘four segments’ PSF, also two parameter varying measurements are necessary for astigmatism aberration, that is rotating the PSF to find the polar orientation of present astigmatism and variation of compensating bias Zernike polynomial (at that rotational orientation).

Advantages in the latter testing are seen for the case that there is no astigmatism present in the system. A single test rotation of the PM will result in a constant (close to zero) signal making a second, amplitude determining measurement step redundant, thus shortening the whole test procedure. The conventional method, however, requires two measuring steps for astigmatism, in order to test for both linear independent versions.

Furthermore, as the orientation of the aberration mode is determined first, the subsequent amplitude measurement ‘sees’ the maximum possible astigmatism signal. In the conventional method, the astigmatism signal will be shared between the two linearly independent astigmatism modes, in average resulting in a decreased signal to noise ratio.

Concerning the exclusiveness of presented PMs to classical aberration modes, it is important to note that indeed, the central intensity of the ‘four segments’ PSF is not exclusively sensitive to primary astigmatism. It rather forms central intensity whenever deviation in the wavefront shows a twofold rotational symmetry that effectively disturbs the π phase difference between neighbouring segments imprinted into the wavefront. In a microscope, where system aberration originates in imperfect lens, mirror, and SLM surfaces, aberration is expected to be smooth and rotational twofold symmetric aberration is likely well approximated to the first order by the Zernike polynomials representing astigmatism. Accordingly, the ‘six segments’ PM is sensitive to aberration of threefold rotational symmetry and the ‘split bullseye’ PM will produce residual central PSF intensity if phase values rise across the pupil in a nonlinear way. This obviously limits the use of binary PMs to systems where low-order aberrations describable by smooth phase functions are dominating.

More general, the three binary PMs are basically ‘flattened’ versions of their corresponding aberration mode. From this similarity an orthogonality between the three binary masks can be deduced. An expansion into Zernike modes (Supplementary Fig. S3) shows, however, that orthogonality to the modes of Zernike is given only for the low order modes of primary astigmatism, trefoil and coma. Higher modes showing similar azimuthal symmetry also cause filling of the central PSF intensity minimum, to a lower extend however. The ‘four segments’ PM shows for example Zernike components (with decreasing amplitude) of secondary astigmatism and sixfoil. This underlines again that the binary PMs may be problematic when sensing complex wavefronts. They are rather suitable for optical systems that benefit already from basic correction of the low order Zernike modes. Concerning the maximal amplitude of aberration accessible by the binary PM method it is noted that an aberration of one wavelength or more will distort the PSF heavily, preventing unambiguous characterization of the wavefront.

### Proof of principle experiments

**Figure 5:**
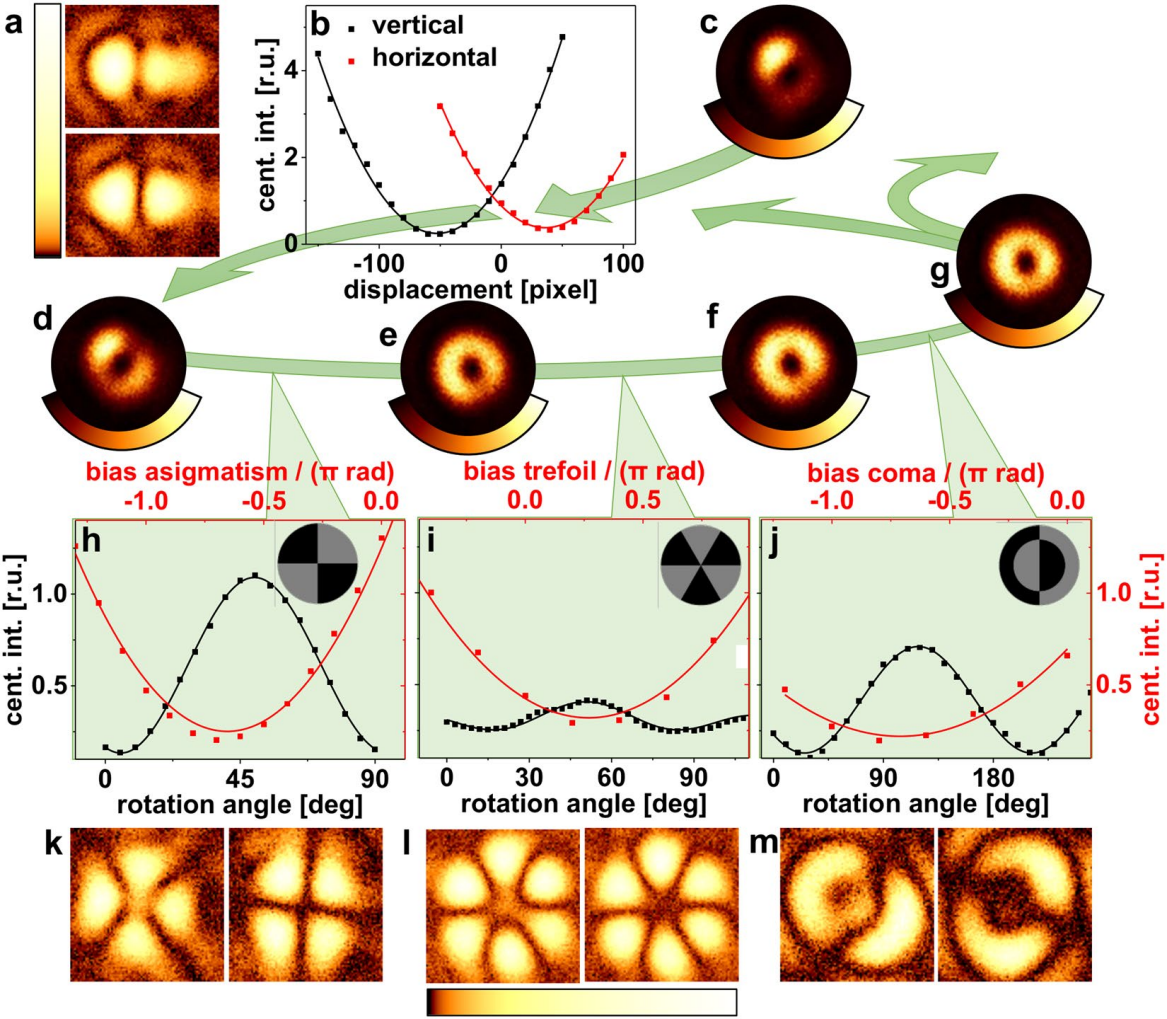
Scheme of stepwise aberration correction by quantifying PM misalignment and wavefront aberrations using specific PMs. (**a**) split PSF for misaligned and well-aligned PM, (**b**) central PSF intensity in (**a**) for varying PM-misplacement, (**c**–**g**) ‘doughnut’ mode after each step of optimization to visualize the progress of aberration correction, (**h**,**k**) central intensity of ‘four segments’ PSF for astigmatism sensing, (**i**,**l**) central intensity of ‘six segments’ PSF for trefoil sensing, and (**j**,**m**) central intensity of ‘split bullseye’ PSF for coma sensing. PSFs from the ‘split’ PM (**a**), and the ‘four segments‘, ‘six segments’, and ‘split bullseye’ PMs (**k**–**m**) are shown in a logarithmic intensity representation to emphasize the disappearance of the central residual intensity by either rotation of the PM (**k**, **m**) or by application of compensating Zernike modes (**l**).

We used binary PMs for beam alignment and system aberration correction in our STED microscope. The proposed adaptive optics scheme is summarized in Fig. 5. An isolated point-like light source is used to sense the properties of the PSF. One possible choice for a ‘guide star’ often used in microscopy is an isolated fluorescent nanobead^6,16^. We have used gold nanobeads with 80 nm in diameter (BBI Solutions, Cardiff, UK) attached to a coverslip and embedded in Mowiol 4–88^25^ for the following proof of principle experiments. Laser light focused by the objective lens is scattered back, collected by the same lens, and detected to either visualize the PSF being scanned over the bead or to probe the central PSF intensity. Corresponding images of phase-modulated focal light intensities are shown in Fig. 5(a,c–g,k–m). Central PSF intensities are given as function of displacement (Fig. 5b) or rotational angle of the sensing PM and amount of additional bias aberration (Fig. 5h–j).

The doughnut-like PSFs (Fig. 5c–g) of the focussed vortex beam are not part of the proposed adaptive optics procedure. They are shown in Fig. 5 only, to qualitatively visualize the stepwise improvement of the optical system. For PSF imaging, the confocal pinhole was removed to circumvent suppression of lateral side lobes.

Having centred the PSF to the isolated nanobead, the first step in the PSF optimization routine is to display the ‘split screen’ PM on the SLM and vary its position on the display, while measuring the intensity of scattered light. Data of corresponding intensity measurements of a vertically and horizontally split PM with the phase border being shifted in steps of 10 pixels are presented in Fig. 5b. The phase border positions resulting in intensity minima give the optimal pixel coordinates, where STED PMs on the SLM should be centred. As a result of an improved alignment of the vortex PM to the STED laser beam, radial symmetry of the ‘doughnut’ PSF increases considerably (Fig. 5c and d).

Light intensity measurements, while firstly rotating the ‘four segments’ PM and secondly varying the bias amplitude of astigmatism at the angle of maximal signal, are given in Fig. 5h. In typical practice we apply about 10 PMs, each for orientation and amplitude sensing of the three considered Zernike modes, that are addressed to the SLM by an ‘slide show player’. In general, the maximum respectively minimum feedback signal could also be determined from only three PM measurements per degree of freedom and least square fitting.

Two focal PSF images for the rotation angles of highest and lowest central signal, respectively, (Fig. 5k) confirm the disappearance of central intensity as predicted by the simulations (see Fig. 2a–e). The quality of the ‘doughnut’ PSF, resulting from the astigmatism-corrected vortex phase (Fig. 5e), is further increased.

Figure 5i and l illustrate the application of the ‘six segments’ PM for measurement of trefoil aberration. The shown PSFs were measured at the same rotational angle of the PM, but with different amounts of bias trefoil for expansion and compensation of wavefront distortions, respectively, leaving the right-hand side PSF image without central intensity. Intensity data for coma sensing are presented in Fig. 5j and example images of involved PSFs are shown in Fig. 5m.

Finally, a well-shaped ‘doughnut’ PSF is achieved after one iteration of aberration correction for low-order Zernike modes (Fig. 5g). A second iteration loop to counter small cross-sensitivities of the used PMs was not necessary in daily practice, since it did not give significant improvement. The high quality of the doughnut in Fig. 5g is in accordance with the observation that aberration in microscopic systems is often dominated by low-order Zernike modes^12^. A perfectly symmetric doughnut shape would probably require the consideration of high-order modes.

### STED performance

**Figure 6:**
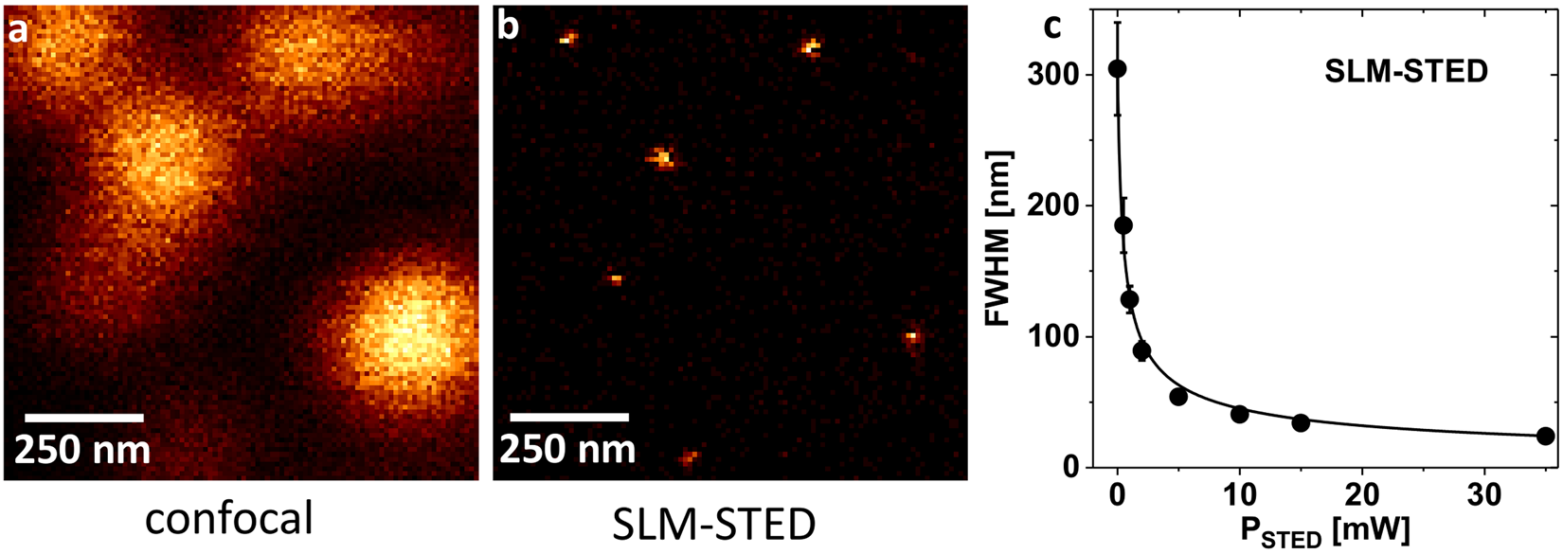
Proof of principle SLM-STED experiment. (**a**) Crimson fluorescence beads (diameter of 20 nm) were imaged in confocal and (**b**) in STED mode at an average STED power of 35 mW (at a repetition rate of 2.5 MHz) with a retrofitted confocal microscope. (**c**) The lateral resolution as determined by averaging the FWHM values of single nanobeads (*N* ≈ 20) at various STED laser powers together with the fit to FWHM = FWHM_confocal_ × (1 + *P*_STED_/*P*_sat_)^−0.5^.

As a final test for the achieved STED beam quality, the corrected ‘doughnut’ PSF was applied for STED imaging. To determine the microscope’s spatial resolution, Crimson fluorescence beads (Life Technologies, Darmstadt, Germany) with a diameter of 20 nm were immobilized on a coverslip pre-treated with 0.1% poly-L-lysine and embedded in Mowiol 4–88^25^. Such fluorescent nanobeads could be used as an almost aberration free sample. STED images (Fig. 6) were recorded and the FWHM diameter of isolated spots were determined by fitting with 2D-Lorentzian peak function. Only peaks in a defined intensity interval were evaluated, to avoid clusters of several fluorescent nanobeads appearing with considerably increased diameter and higher brightness. The determined FWHM values shown in Fig. 6c may for this discrimination of larger particles be seen as a lower threshold of the optical STED resolution.

With an available average STED laser power of *P*_STED_ = 35 mW (at a repetition rate of 2.5 MHz) single fluorescent nanobeads could be imaged with the SLM-STED setup to an average FWHM spot width of 24 ± 4 nm. The STED laser power dependence of the achievable resolution was well described by the one over square root dependency predicted for STED microscopy (Fig. 6c).

## Conclusions

By mathematical modelling and in experiments we have investigated the use of binary phase masks for the optimization of high NA imaging systems equipped with a spatial light modulator. Basic binary PMs were found to create PSFs that allow direct evaluation of major disturbances by inspection of the focal intensity distribution. If greyscale level PMs exist that show a better specificity or sensitivity to chosen aberration modes, was not investigated in this study.

Applied in SLM-based STED laser beam shaping, our approach avoids complex image evaluation that requires trained staff or sophisticated software solutions by relying on the obvious specificity of the shaped PSFs. We have demonstrated the applicability of the proposed PMs for modal wavefront sensing in a confocal microscope retrofitted with an STED beam and a SLM and have performed fluorescence imaging of fluorescence beads with lateral resolution of ~25 nm. Discussed advantages of the proposed PSFs for the estimation of low-order aberrations include a better detectability of signal modulation on top of a close to zero background of the central intensity valley, all sensor PSFs have in common. A main drawback is the limitation to smooth low-order aberrations up to Zernike polynomials of third order.

Also, apart from microscopy, the presented PMs may show helpful for SLM applications concerning alignment and coarse aberration testing, as apart from the SLM only a point detector or a camera are necessary, to realize the proposed optical aberration and alignment testing.

The proposed binary PMs might also find application in phase retrieval methods based on the Gerchberg-Saxton algorithm. We suspect that expansion of the investigated wavefront by these PMs may have a beneficial effect on the convergence of the algorithm if different PSFs, each with specific sensitivity to different aberration symmetries, are given as input. But we have not further investigated this idea.

## Methods

### Focal field simulations

Focal field calculations were applied to investigate the effects of primary classical aberrations on the PSFs created by different binary PMs upon focusing the corresponding circularly polarized pupil function with a high NA objective. Thus, phase modulation experiments could be verified under strictly controlled conditions regarding misalignment and aberration. To account for significant contributions of polarization effects when focusing with a high NA objective lens, the vectorial diffraction theory of Richards and Wolf ^26,27^ was applied, to compute focal fields and intensity distributions. 2D-Fast Fourier Transform (FFT) operations allow for efficient computation of vectorial Debye integrals for arbitrary polarization states of the pupil function^14,28^ and were used in the implementation of Boruah and Neil^14^. Comparison of simulation results with experimental data confirmed the correctness of our implementation.

All focal intensity calculations shown in this work were performed for a circularly polarized entrance pupil, for a wavelength of 766 nm, NA of 1.45, and the refractive index was taken to be *n* = 1.5. Equidistant sampling with 96 sample points over the aperture diameter and zero-padding to a total of 1024 FFT sample points per dimension were applied resulting in a step width of *Δx* = *Δy* = 24.8 nm in the calculated PSFs. According to the measures in our experimental setup, the lateral laser intensity distribution was approximated by a 2D Gaussian function with a full width half maximum (FWHM) diameter filling 58% of the aperture. Assuming a Gaussian beam of width *w* centred to the circular aperture, the final pupil functions, *P*, including the binary phase pattern (*ϕ* ^PM^) and a Zernike aberration mode (*Z*_j_) with peak-to-valley bias amplitude *a*_*j*_ were implemented as:

1 $$P=aperture({k}_{x},\,{k}_{y})\times Exp[-({k}_{x}^{2}+{k}_{y}^{2})/{w}^{2}]\times Exp[i({\sum }_{j}{a}_{j}{Z}_{j}({k}_{x},{k}_{y})+{\varphi }^{PM}({k}_{x},\,{k}_{y}))]$$

The Zernike polynomials and series of them are frequently used to describe optical aberration mathematically. However, when used in microscopic context with high NA and non-uniform pupil functions, they are not strictly orthogonal anymore and distortion of the wavefront by some modes as e.g. coma result in an additional lateral shift of the focus^6^. For moderate aberration, these shifts are in first approximation linear and were taken into account for by adding shift-compensating amounts of tilt to the coma mode.

### Experimental setup

As a platform for the realization of SLM-STED microscopy and sensorless wavefront measurements with binary PMs we have chosen the commercial confocal microscope system MicroTime200 (PicoQuant, Berlin, Germany), which we had already retrofitted with STED modality utilizing a static beam shaping device in the past^25,29^.

For STED depletion, a ps-pulsed 765 nm STED laser (VisIR-765, pulse length > 300 ps, P_max_ = 1.5 W, PicoQuant) was spatially filtered by coupling into a high-power, polarization maintaining optical fibre and guided to the centre of the SLM-display (PLUTO-NIR-015-C, HOLOEYE Photonics, Berlin, Germany) at an incident angle of 7°. The phase-modulated wavefront passed a Glan-Thompson polarizer and was brought to an exact lateral overlap with the excitation laser beam (ps-pulsed 635 nm laser diode, PicoQuant) by a shortpass dichroic mirror (725dcspxr, Chroma, Olching, Germany). An achromatic λ/4-plate changed the polarization state to circular and the dual-band dichroic mirror (zt635/766rpc, AHF Analysentechnik, Tübingen, Germany) separated excitation light and STED light from the fluorescence signal in a confocal detection scheme (pinhole diameter of 50 µm).

**Figure 7:**
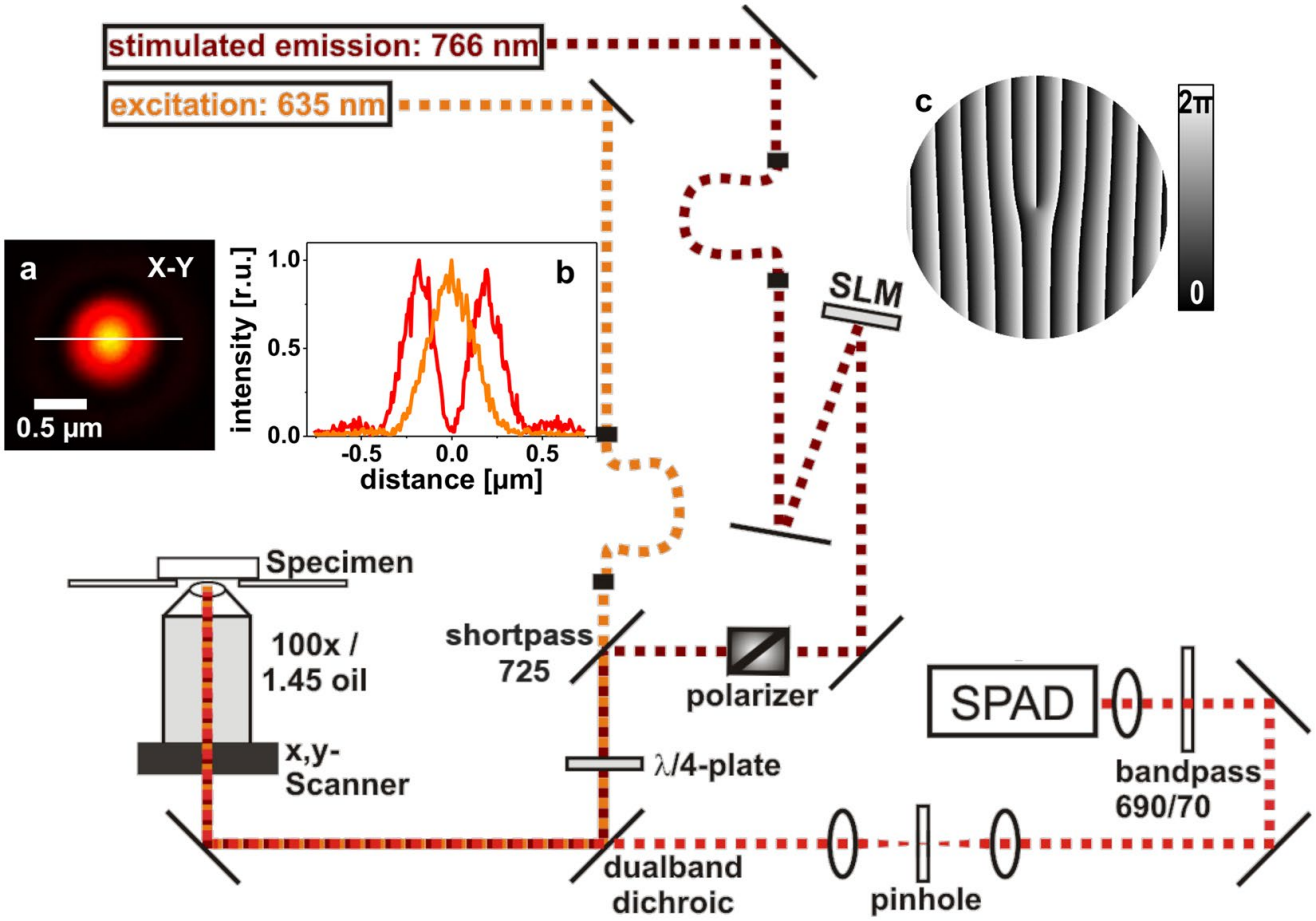
Scheme of the confocal microscope expanded by the SLM-STED components. Excitation and STED laser beams were spatially filtered, collimated, and combined by a dichroic mirror. Confocal detection was realized by a 50 µm pinhole and a single photon avalanche photodiode (SPAD). Polarization state of light from the spatial light modulator (SLM) is changed to circular by a Glan-Thompson polarizer and an achromatic λ/4-plate. (**a**) excitation (orange) and STED (red) focal intensity distributions. (**b**) Profile plot of normalized laser intensities along white line in (**a**). (**c**) Example for PM programmed into the SLM.

Image acquisition was realized by piezo-scanning of the 100× oil immersion objective (Nikon CFI P-Apo 100× Lambda Oil, NA 1.45). Fluorescence light was detected in the spectral range of 655–725 nm (bandpass 690/70 detection filter) with ps-time resolution by a single photon avalanche diode (SPAD) in combination with a time-correlated single-photon counting (TCSPC) module (PicoHarp300, PicoQuant). The overall layout of the system (Fig. 7) was determined by the confocal system that should be expanded without changes to its main optical path. Otherwise an optical layout with the STED-beam shaping SLM closer to the objective lens would probably be of benefit.

The SLM was gamma-corrected to guarantee a linear phase shift response in the range of 0-2π rad to video grey levels in the range of 0–255. A corresponding lookup table was determined in advance with a two-beam interferometer setup^30^.

### Phase mask definition

The vortex PM used for the creation of the doughnut-shaped focal STED intensity distribution (Fig. 7a) can be represented by the transmission function

2 $$P{M}^{Vortex}=\exp (i\phi )$$

where *φ* is the azimuth of the polar coordinate system describing the pupil plane. In Cartesian coordinates, the corresponding phase function *ϕ* ^Vortex^ applied on the SLM is readily described by the arctan2 function, a variation of the arctangent function, which takes the quadrants of the coordinate system into account to avoid ambiguous results in coordinate transformation.

3 $${\varphi }^{Vortex}({k}_{x},{k}_{y})=\phi ({k}_{x},{k}_{y})=\arctan 2({k}_{y},{k}_{x})$$

Additionally, tilt in form of a linear phase ramp (*ϕ* ^Tilt^(*k*_x_,*k*_y_) = 18 × 2π × *k*_x_) was added to the vortex, resulting in the ‘fork’-phase pattern after phase wrapping to 2π (Fig. 7c). The linear phase ramp, forming a blazed grating after wrapping, diffracts the focal light intensity by a few micrometres off the optical axis and thus, prevents that residual light reflected from the SLM without proper phase modulation fills up the intensity minimum of the STED ‘doughnut’^5^. A linear combination of Zernike polynomials is finally added to the ‘fork’ PM, in order to compensate for aberration in the optical system, according to equation (1).

The binary PMs proposed for misalignment and aberration sensing are mathematically described below. Starting from a two-dimensional matrix coordinate mesh with *k*_*x*_- and *k*_*y*_-dimension in the range [−1.0;1.0], a ‘half space’ phase function with vertical π phase step (Fig. 1a inset) is defined by

4 $${\varphi }^{split}=\{\begin{array}{cc}\pi  & {k}_{x}\ge 0\\ 0 & {k}_{x} < 0\end{array}.$$

For a horizontal split, *k*_x_ in equation (4) is substituted by *k*_y_, accordingly. A ‘four segments’ PM (Fig. 2a inset) was defined by the two-dimensional phase function

5 $${\varphi }^{4Seg}=\{\begin{array}{cc}\pi  & ({k}_{x}\times {k}_{y}\ge 0)\\ 0 & ({k}_{x}\times {k}_{y} < 0)\end{array}.$$

A ‘split bullseye’ PM (Fig. 3a inset) that is proposed for coma sensing here, was calculated as combination of a circle aperture of radius *R*:

6 $${\varphi }^{Circ}=\{\begin{array}{cc}\pi  & ({k}_{x}^{2}+{k}_{y}^{2}\le {R}^{2})\\ 0 & ({k}_{x}^{2}+{k}_{y}^{2} > {R}^{2})\end{array}$$

and the ‘half space’ according to:

7 $${\varphi }^{SBE}=({\varphi }^{Circ}+{\varphi }^{split})\mathrm{mod}\,2\pi ,$$

where mod 2π is the modulo function giving the remainder of division by 2π. For a flat-top laser intensity profile a good radius *R* choice is close to 0.8 times the pupil radius to coincide roughly with the outer zero of the coma polynomial. For simulations and experiments a radius of 0.6 was applied to take into account the Gaussian intensity profile in our system.

A binary ‘six-segments’ PM with circularly alternating phase values of zero and π (Fig. 5a inset) can be described by the polar angle coordinate *φ* again, as:

8 $${\varphi }^{6Seg}=\{\begin{array}{cc}\pi  & 0\le \phi  < \pi \,/3\,or\,2\pi \,/3\le \phi  < \pi \,or\,4\pi \,/3\le \phi  < 5\pi /\,3)\\ 0 & \pi /3\le \phi  < 2\pi /3\,or\,\pi \le \phi  < 4\pi \,/3\,or\,5\pi \,/3\le \phi  < 2\pi )\end{array}.$$

### Data Availability

The datasets generated during and/or analysed during the current study are available from the corresponding authors on reasonable request.

## Electronic supplementary material

Supplementary Information
