# Supplementary material for: Binary phase masks for easy system alignment and basic aberration sensing with spatial light modulators in STED microscopy
Source: Sci Rep. 2017 Nov 16;7:15699. doi: 10.1038/s41598-017-15967-5 (PMC5691043; doi:10.1038/s41598-017-15967-5)
Supplement: Supplementary file 1 — Supplementary Information [file 41598_2017_15967_MOESM1_ESM.pdf]

Supplementary Information

Binary phase masks for easy system alignment and basic aberration sensing with spatial light modulators in STED microscopy

A. Klauss[1,*], F. Conrad[1], and C. Hille[1,2]

[1]University of Potsdam, Institute of Chemistry, Potsdam, D-14476, Germany
[2]hille@uni-potsdam.de
*aklauss@uni-potsdam.de

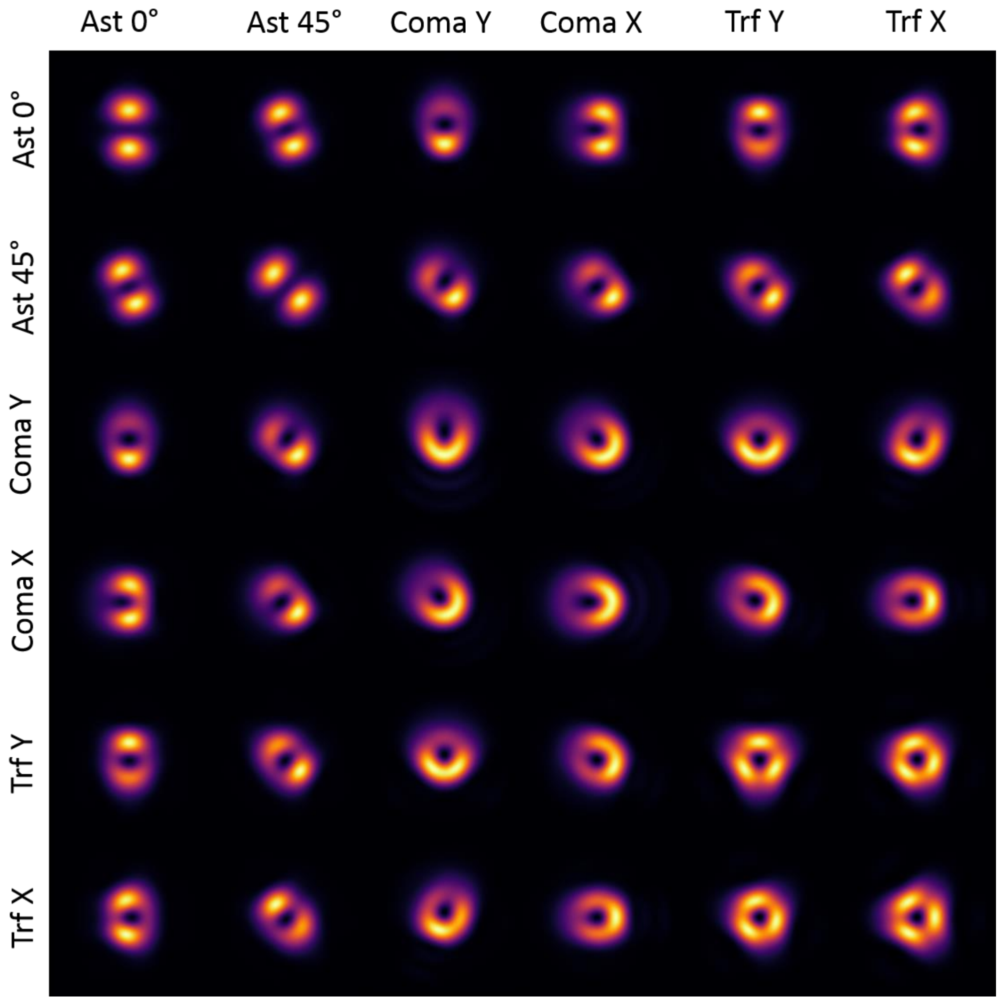

Figure S1. Simulated PSFs of circularly polarized vortex beams aberrated by different combinations of the low order Zernike modes of amplitude $\phi = 0.4$ rad, representing astigmatism (Ast), coma (Coma), and trefoil (Trf). On the main diagonal the effect of only one aberration mode on the focal intensity distribution becomes visible. Off the diagonal the combined effect of two different Zernike aberrations are shown.

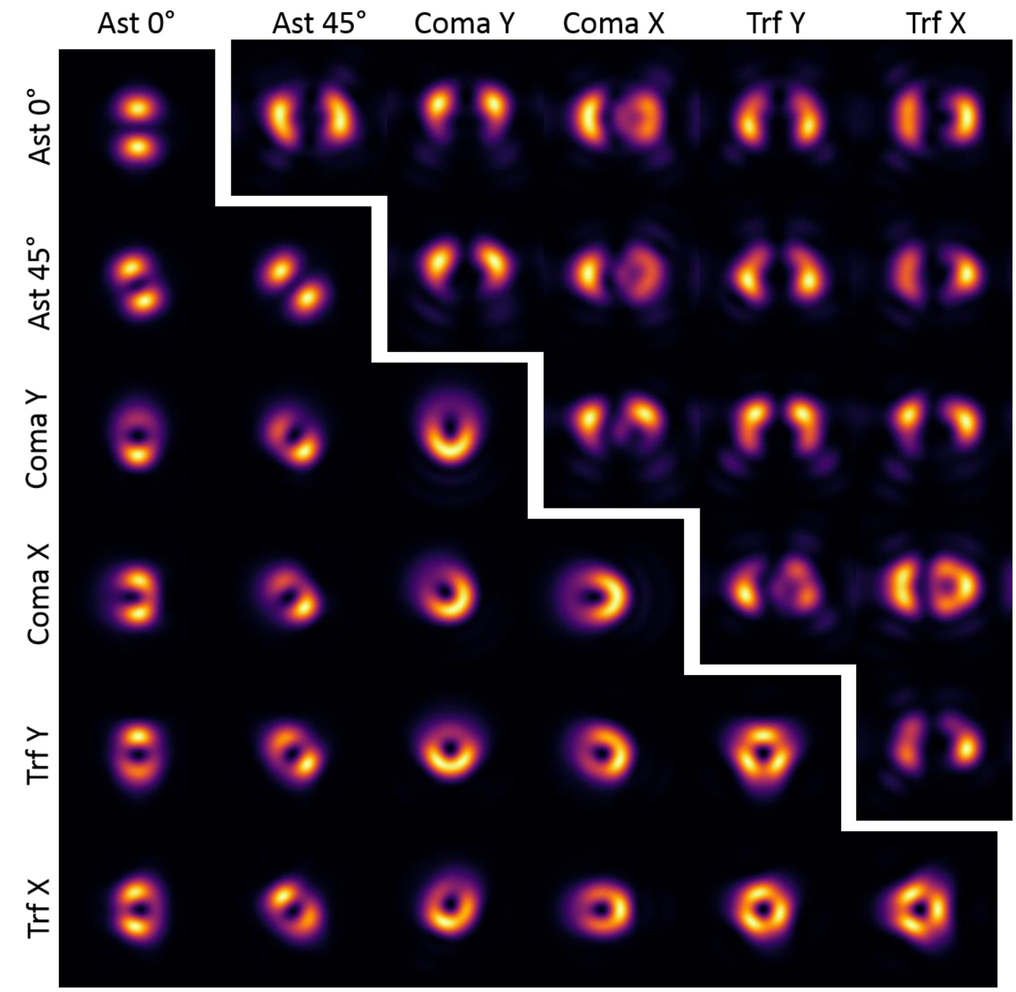

Figure S2. Simulated PSFs of a vortex beam and a beam phase-modulated by a binary pattern of the shape of a ‘split bullseye’. Both beams are aberrated by different combinations of two Zernike modes of amplitude ϕ = 0.4 rad. The PSF of the binary ‘split bullseye’ phase mask shows specific sensitivity to coma aberration in ‘X-orientation’ clearly visible in the central residual intensity at those five PSFs above the main diagonal that contained coma in that direction (Coma X).

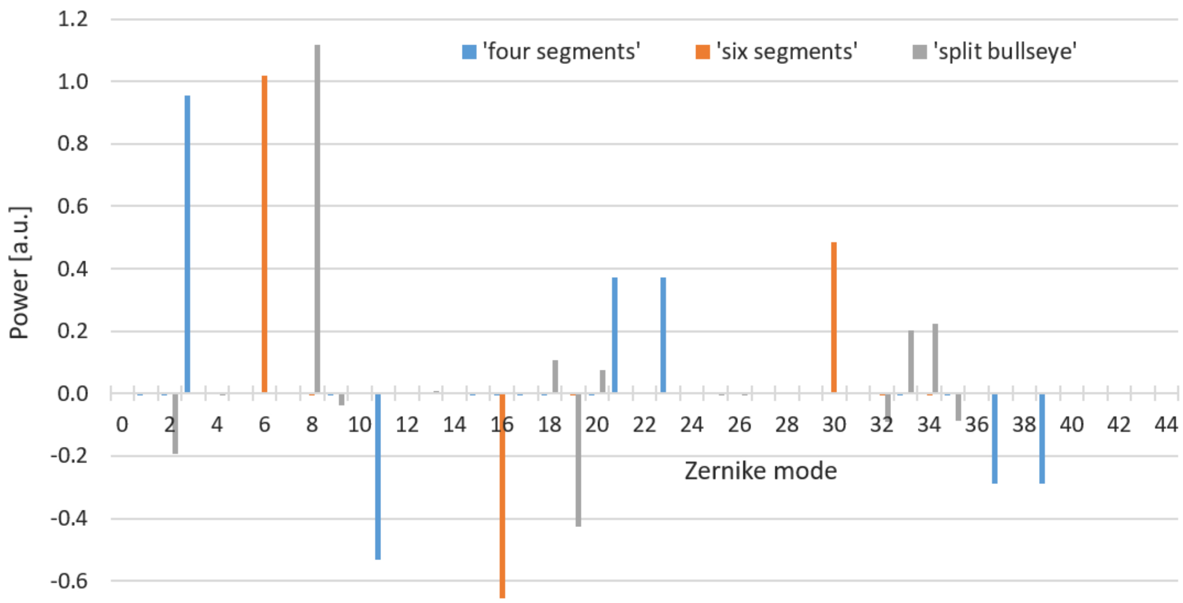

n	m	j		
0	0	0		Piston
1	-1	1		Tip
1	1	2		Tilt
2	-2	3		Astigmatism 45°
2	0	4		Defocus
2	2	5		Astigmatism 0°
3	-3	6		Trefoil 0°
3	-1	7		Coma 0°
3	1	8		Coma 90°
3	3	9		Trefoil 30°
4	-4	10		Tetrafoil

n	m	j		
4	-2	11		2nd Astigmatism 45°
4	0	12		Spherical
4	2	13		2nd Astigmatism 0°
4	4	14		Tetrafoil
5	-5	15		Pentafoil
5	-3	16		2nd Trefoil 0°
5	-1	17		2nd Coma 0°
5	1	18		2nd Coma 90°
5	3	19		2nd Trefoil 30°
5	5	20		Pentafoil
...	...	...		...

Figure S3. Zernike expansion of the binary ‘four segments’, ‘six segments’, and ‘split bullseye’ phase masks. Numeration of the Zernike polynomials Z_n^m by a single parameter *j* is illustrated in lower tables. The highest component for each phase mask is found to be the Zernike mode the particular binary phase mask is aiming for. To a lower extend also higher Zernike polynomials with similar azimuthal symmetry appear in the expansions. The inner radius parameter *R* for the ‘split bullseye’ phase mask was set to 0.8 times the radius of the pupil for the expansion, as intensity distributions different from flat top are neglected in the expansion.
